# The Transition from Gastric Intestinal Metaplasia to Gastric Cancer Involves *POPDC1* and *POPDC3* Downregulation

**DOI:** 10.3390/ijms22105359

**Published:** 2021-05-19

**Authors:** Rachel Gingold-Belfer, Gania Kessler-Icekson, Sara Morgenstern, Lea Rath-Wolfson, Romy Zemel, Doron Boltin, Zohar Levi, Michal Herman-Edelstein

**Affiliations:** 1 The Felsenstein Medical Research Center, Rabin Medical Center, Petah Tikva 4941492, Israel; icekson@tauex.tau.ac.il (G.K.-I.); zemel@tauex.tau.ac.il (R.Z.); Michalh6@clalit.org.il (M.H.-E.); 2 Rabin Medical Center, Gastroenterology Division, Petah Tikva 4941492, Israel; dboltin@gmail.com (D.B.); Zohar.levi.gastroenterology@gmail.com (Z.L.); 3 Sackler Faculty of Medicine, Tel Aviv University, Tel Aviv 6997801, Israel; leawolfson@gmail.com; 4 Rabin Medical Center, Department of Pathology, Petah Tikva 4941492, Israel; saramo@clalit.org.il; 5 Rabin Medical Center, Department of Nephrology, Petah Tikva 4937211, Israel

**Keywords:** gastric intestinal metaplasia, gastric cancer, *POPDC1* (*BVES*), *POPDC3*

## Abstract

Intestinal metaplasia (IM) is an intermediate step in the progression from premalignant to malignant stages of gastric cancer (GC). The Popeye domain containing (*POPDC*) gene family encodes three transmembrane proteins, POPDC1, POPDC2, and POPDC3, initially described in muscles and later in epithelial and other cells, where they function in cell–cell interaction, and cell migration. *POPDC1* and *POPDC3* downregulation was described in several tumors, including colon and gastric cancers. We questioned whether IM-to-GC transition involves *POPDC* gene dysregulation. Gastric endoscopic biopsies of normal, IM, and GC patients were examined for expression levels of *POPDC1-3* and several suggested IM biomarkers, using immunohistochemistry and qPCR. Immunostaining indicated lower POPDC1 and POPDC3 labeling in IM compared with normal tissues. Significantly lower *POPDC1* and *POPDC3* mRNA levels were measured in IM and GC biopsies and in GC-derived cell lines. The reduction in focal IM was smaller than in extensive IM that resembled GC tissues. *POPDC1* and *POPDC3* transcript levels were highly correlated with each other and inversely correlated with *LGR5*, *OLFM4*, *CDX2*, and several mucin transcripts. The association of *POPDC1* and *POPDC3* downregulation with IM-to-GC transition implicates a role in tumor suppression and highlights them as potential biomarkers for GC progression and prospective treatment targets.

## 1. Introduction

Recent global statistics rank gastric cancer (GC) fifth for incidence and third for cancer-related mortality worldwide and a major world health concern [1]. The disease is frequently associated with poor prognosis due to late detection when curative treatment is limited [1,2]. GC evolves through slow progressing multistep alterations that include chronic gastritis, gastric intestinal metaplasia (IM), dysplasia, and early and advanced gastric cancer. IM is characterized by replacement of normal gastric mucosa by intestinal epithelium in response to chronic gastric inflammation. IM is present in approximately one-fourth (19–30%) of individuals worldwide and is characterized by gastric lesions of small intestinal-specific phenotype where goblet cells and enterocytes replace gastric mucosa cells [3,4,5]. The fact that most IM cases do not progress into GC indicates that the transition from gastric pre-malignancy to malignancy is complex and involves diverse factors. Available information associates the de novo expression of caudal homeobox transcription factors 1 and 2 (*CDX1*, *CDX2*) and mucin 2 (*MUC2*) with IM development [6,7,8,9,10], and the increased expression of intestinal stem cell (ISC) markers, including *LGR5*, *OLFM4*, and *EPHB2*, with IM-related gastric tumorigenesis [6,11]. The identification of additional players in the process remains of major importance for the understanding of IM to GC transition and the introduction of suitable biomarkers for early detection and improved therapeutic approaches.

The Popeye domain containing (*POPDC*) gene family comprises three transmembrane cyclic AMP effector proteins encoding POPDC1 (also named blood vessel epicardial substance, BVES), POPDC2 and POPDC3. POPDC proteins, originally discovered in muscles, are present in several cell types including epithelial cells [12,13]. The POPDC proteins play an important role in striated muscle homeostasis, such as skeletal muscle regeneration and the control of heart rhythm, heart stress signaling, and heart cell survival [14,15,16,17]. In epithelial cells, POPDC proteins function in cell-cell interaction and affect cell adhesion, proliferation and migration [15,18]. In several tissues including the stomach, downregulation of *POPDC1* and *POPDC3* expression via DNA promoter hypermethylation has been shown to enhance tumorigenesis and promote cell proliferation, migration, invasion, and metastasis that correlate with disease progression and clinical outcome [19,20,21,22]. *POPDC1* and *POPDC3* likely function as tumor suppressors and the reported inverse relationship between *POPDC1* levels and *c-Myc* expression and *Wnt* signaling may link *POPDC1* underexpression to intestinal stem cell programming and malignant tumor growth [23,24,25,26,27,28,29,30]. We questioned whether and how *POPDC* genes are reprogrammed upon the transition from IM to GC and investigated their expression profile relative to genes of known association with gastric tumorigenic alterations.

## 2. Results

### 2.1. Details of Tissue Specimens

A cohort of 80 archived endoscopic antral gastric biopsies was recruited at the Rabin Medical Center Pathology Department that included gastric IM (*N* = 40), gastric cancer (*N* = 20) and gastric normal tissues (*N* = 20) as assessed by an expert gastro-pathologist, based on H&E-staining. The IM specimens were categorized as focal IM, when IM morphology occupied less than 30% of the biopsy area (*N* = 22) or extensive IM when biopsy area was greater (*N* = 18). The normal biopsies were of patients admitted to gastroscopy according to clinical indications, such as iron deficiency anemia, weight loss, or epigastric pain investigation. The cohort details including age, gender, and histology are summarized in Table 1. Note that the mean age of GC patients was 10 years higher than that of patients with gastric IM and with normal histology.

Of the 80-patient cohort, 17 focal IM patients (77.3%), 14 extensive IM patients (77.7%), and 8 normal tissue patients (40%) underwent endoscopic surveillance 1–2 years after the index gastroscopy. Among these patients, 32% of the focal and the extensive IM cases regressed to chronic atrophic gastritis. One focal IM patient progressed to extensive IM and 2 extensive IM patients reversed to focal IM. A single patient with extensive IM developed GC stage 1 and underwent early gastrectomy. Two patients who had extensive IM with high grade dysplasia underwent preventive partial gastrectomy. None of the normal gastric tissue patients developed IM or GC. In this study, we did not analyze any of the endoscopic follow-up tissues.

### 2.2. POPDC Protein Distribution

**Figure 1:**
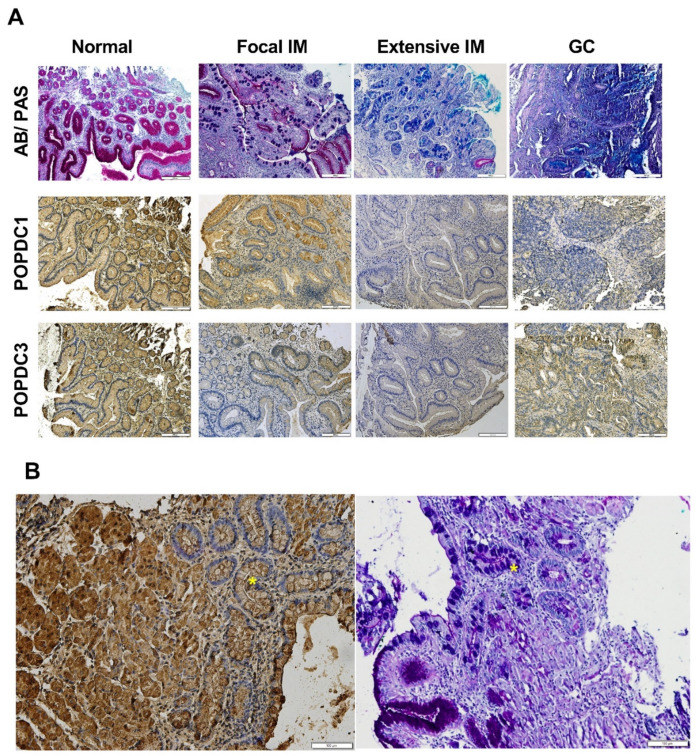
Representative biopsy microsections. (**A**) The four tissue categories stained for mucins (AB/PAS, purple) and immunostained for *POPDC1* and *POPDC3*. The mucus in goblet cells in IM stain magenta for neutral mucins and bright blue for acid mucin. The brown color represents protein immunolabeling of *POPDC1* and *POPDC3*. Acidic mucins appear in blue and neutral mucins in red; mixed mucins appear purple. The images shown are not serially overlapping. Note reduced labeling intensity of POPDC1 and POPDC3 in the IM and GC tissues. X20 magnification. (**B**) A transitional region from normal phenotype to IM (yellow asterisks). Left, POPDC1 immunolabeling; right, mucin AB/PAS staining. Note typical morphology and reduced POPDC1 labeling in the IM region. The bar indicates 100 µm.

Immunohistochemical (IHC) evaluation of *POPDC1* and *POPDC3* indicated lower labeling in the luminal surface of gastric glands of IM, compared with normal gastric tissues and normal adjacent tissues (Figure 1A). In all cases, Alcian blue/Periodic acid-Schiff (AB/PAS) staining was performed that stained both acidic and neutral mucins and delineated gastric tissue morphology and goblet cells in IM. Figure 1B represents a transitional region from normal gastric tissue to IM.

### 2.3. POPDC mRNA Expression

**Figure 2:**
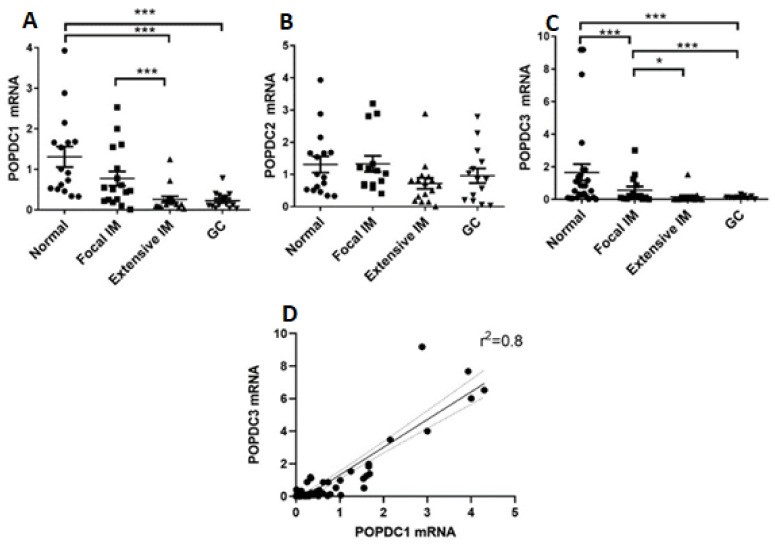
Expression of *POPDC* genes in the four gastric biopsy categories. A (**A**–**C**), Expression levels of *POPDC* genes in normal (●), focal IM (■), extensive IM (▲), and GC (▼) biopsies as measured by qPCR. (**A**) *POPDC1,* (**B**) *POPDC2,* (**C**) *POPDC3.* Values of mRNA scores are in relative quantity (RQ) normalized to *RPLP0*. Mean ± SEM. * *p* < 0.05; *** *p* < 0.001. (**D**) Correlation analysis between *POPDC1* and *POPDC3* in all the IM tissues. *p* < 0.001.

To estimate the expression of *POPDC* genes in the different specimens we extracted RNA from tissue microsections and quantified the relative amounts of *POPDC* mRNA species using qPCR. As shown in Figure 2A–C, the mean levels of *POPDC1* and *POPDC3* transcripts were higher in normal and in focal IM tissues compared with extensive IM and GC tissues that resembled each other. *POPDC3* expression levels in focal IM were significantly lower than in normal tissues. *POPDC2* expression remained essentially unchanged in the different gastric phenotypes. A statistically positive correlation was observed between the transcript levels of *POPDC*1 and *POPDC3* (Figure 2D).

**Figure 3:**
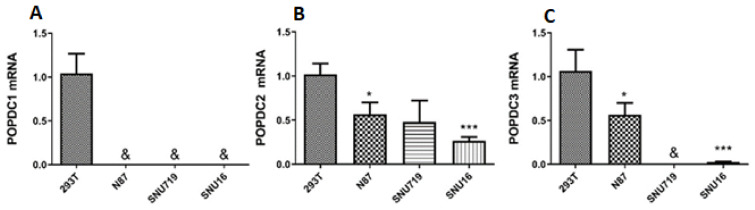
Expression of *POPDC* genes in three GC cell lines. Results are presented as fold difference from 293T reference cells. (**A**) *POPDC1*; (**B**) *POPDC2*; (**C**) *POPDC3*. Mean ± SEM; * *p* < 0.05 vs. 293T cells; *** *p* < 0.001 vs. N87 cells; &, below detection.

We next examined whether the expression levels of *POPDC* genes varied between cell lines established from GC tumors and analyzed three GC cell lines of diverse origins and phenotypes: (1) N87, derived from liver metastasis, well differentiated intestinal type cells displaying in culture an epithelial monolayer [31]; (2) SNU719, derived from a primary tumor, moderately-differentiated gastric adenocarcinoma cells [32]; (3), SNU16, derived from metastatic ascites, poorly-differentiated adenocarcinoma cells growing in culture as non-attached floating cells [31]. In the absence of normal gastric cells, we normalized the transcript values of each *POPDC* gene to the corresponding mean values obtained from 293T cells, a human embryonic kidney-derived cell line that maintains normal epithelial morphology and expresses the three *POPDC* isogenes. The silencing of *POPDC1* in HEK293T cells increased their susceptibility to infection with enteropathogenic bacteria similar to the increased sensitivity of colonic endothelial cells to bacterial infection in *POPDC*1 null mice [27]. As shown in Figure 3, *POPDC1* was practically undetected in any of the three GC cell lines. *POPDC2* mRNA was observed in all the cell lines, yet significantly lower expression levels were measured in the poorly differentiated floating SNU16 cells. *POPDC3* mRNA was absent in the less differentiated SNU719 and SNU16 cell lines, but was evident in the well differentiated N87 intestinal type cells. Namely, *POPDC1*, *POPDC2*, and *POPDC3* are dysregulated differently in the various GC cell lines, partially reflecting the degree of cell differentiation and malignancy.

### 2.4. Expression of Genes Associated with IM and GC Progression

**Figure 4:**
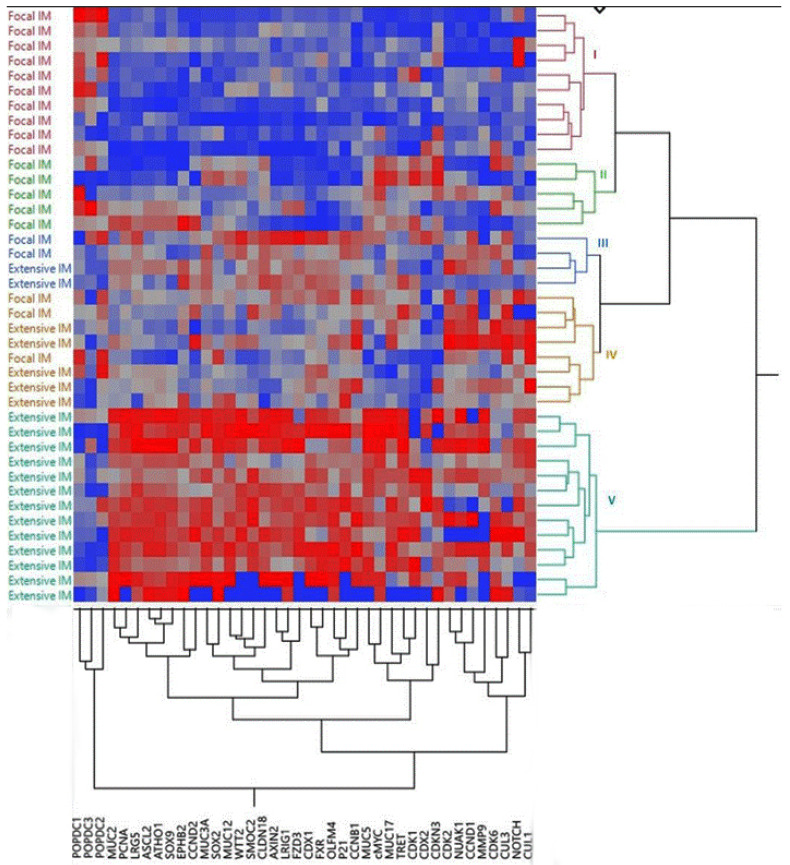
A heatmap of gene expression and bidirectional hierarchical clustering of the IM tissues. Expression values of *POPDC1-3* and a selection of genes associated with IM and GC progression were analyzed. Color codes are from low (blue) to high (red) expression values. Each row represents an individual tissue specimen categorized according to the clinico-histological identification. Each column depicts a single gene as label at the bottom. The bidirectional hierarchical clustering generated two dendrograms: (1) the specimens (right), five main clusters (I-V), of which clusters I, II, comprise focal IM only, cluster V comprises extensive IM only, and clusters III, IV, comprise focal and extensive IM samples. (2) The mRNA species (bottom), five main gene clusters. Note a discrete *POPDC1-3* cluster (bottom left) where *POPDC1* and *POPDC3* segregate out of *POPDC2*.

We compared the expression patterns of *POPDC1-3* with those of genes encoding regulators of cell proliferation, cell cycle, adhesion, migration as well as mucins and stem cell markers, all related to gastric cell growth and malignancy [11]. A heatmap and bidirectional hierarchical clustering of gene expression within the four gastric tissue categories ordered the specimens into several super clusters, which diverged further to smaller clusters assembling each tissue category into several distinct groups along with some category intermixing (not shown). A heatmap of the IM tissues only allocated the specimens into five main clusters of which two clusters (15 samples) grouped focal IM only, one cluster (13 samples) comprised extensive IM only and two additional clusters (a total of 12 samples) included both focal and extensive IM (Figure 4). The analyzed genes assembled into 6 main clusters allocating the *POPDC* transcripts to a distinct three-gene cluster where *POPDC1* and *POPDC3* separated out from *POPDC2* (Figure 4).

Following the exploratory heatmap analysis that discriminated *POPDC* gene expression from almost all the other genes analyzed, we examined further the relationship between *POPDC1* and *POPDC3* expression levels and those of genes representing regulators of proliferation/growth, mucins, and gastric stem cell markers.

### 2.5. Regulators of Transcription

**Figure 5:**
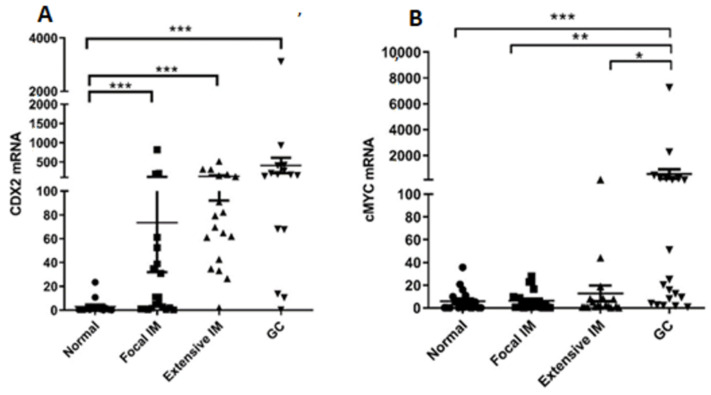
Expression levels of transcription factors: (**A**) CDX2 and (**B**) c-MYC in normal (●), focal IM (■), extensive IM (▲), and GC (▼) gastric biopsies measured by qPCR. * *p* < 0.05; ** *p* < 0.005; *** *p* < 0.001. Mean ± SEM.

Transcript levels of CDX2, a homeobox transcription factor essential for intestinal cell growth and differentiation and a molecular trigger in IM and gastric carcinogenesis, and *c-Myc*, a transcription factor associated with gastric cancer progression, were assessed, and correlation with *POPDC1* and *POPDC3* expression was calculated [8,11,33]. Compared to normal gastric tissues, *CDX2* transcripts were markedly elevated in focal and extensive IM and in GC tissues (Figure 5A). c-Myc transcripts were essentially unchanged in the focal IM, few were elevated in extensive IM, and numerous were significantly elevated in GC tissues (Figure 5B). A statistically significant inverse correlation was found between the transcript levels of *CDX2* and *POPDC1* and *POPDC3* (r = −0.6492, *p* < 0.001 and r = −0.5242, *p* < 0.001, respectively). As for *c-Myc*, an inverse correlation was calculated for *POPDC1* (r = −0.2439, *p* < 0.05) not for *POPDC3*.

### 2.6. Mucins

**Figure 6:**
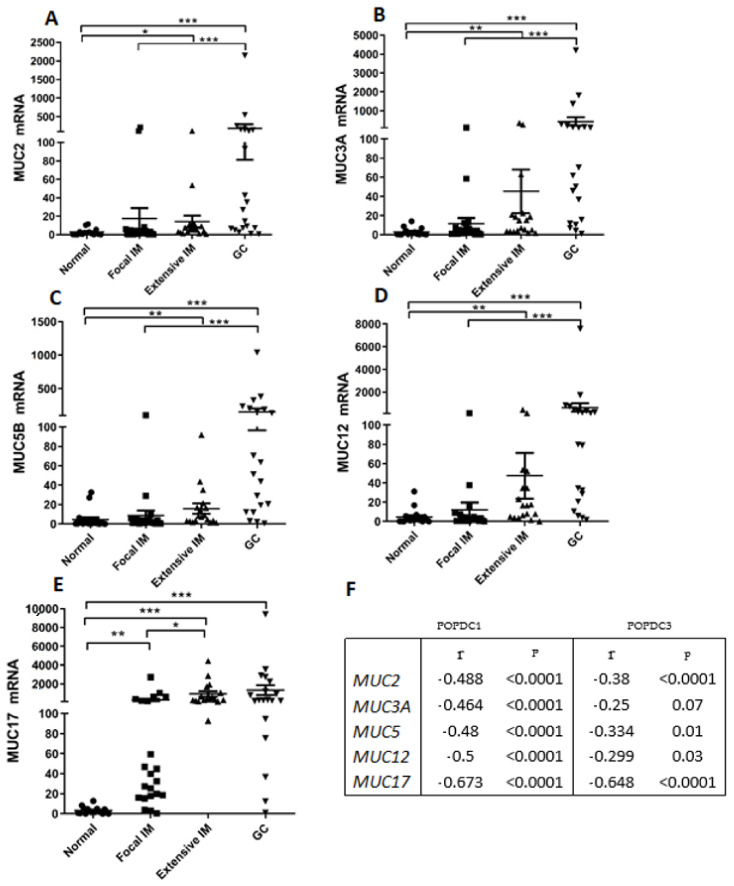
Expression levels of mucin genes and correlation with *POPDC1* and *POPDC3* expression (**A–E**). Summary of mucin transcript quantification. *MUC*, mucin. * *p* < 0.05; ** *p* < 0.005; *** *p* < 0.0001. Mean ± SEM. (**F**)Results of Spearman’s correlation test between the transcript levels of each mucin and *POPDC1* and *POPDC3*.

Expression of several mucins has been linked to IM transdifferentiation and neoplastic transition in the stomach [34,35]. We examined the relationship between *POPDC1* and *POPDC3* expression levels and those of secreted gel-forming (*MUC2*, *MUC5*), secreted non-gel forming (*MUC17*) and membrane bound (*MUC3A, MUC12*) mucins [36]. Compared with normal gastric tissues, all five mucins displayed significantly elevated expression in extensive IM and GC samples with few specimens elevated also in focal IM. *MUC17* was also significantly elevated in focal IM (Figure 6). Correlation analyses with *POPDC1* and *POPDC3* mRNAs indicated statistically significant inverse correlations with the five mucins (Figure 6).

### 2.7. Stem Cell Markers

**Figure 7:**
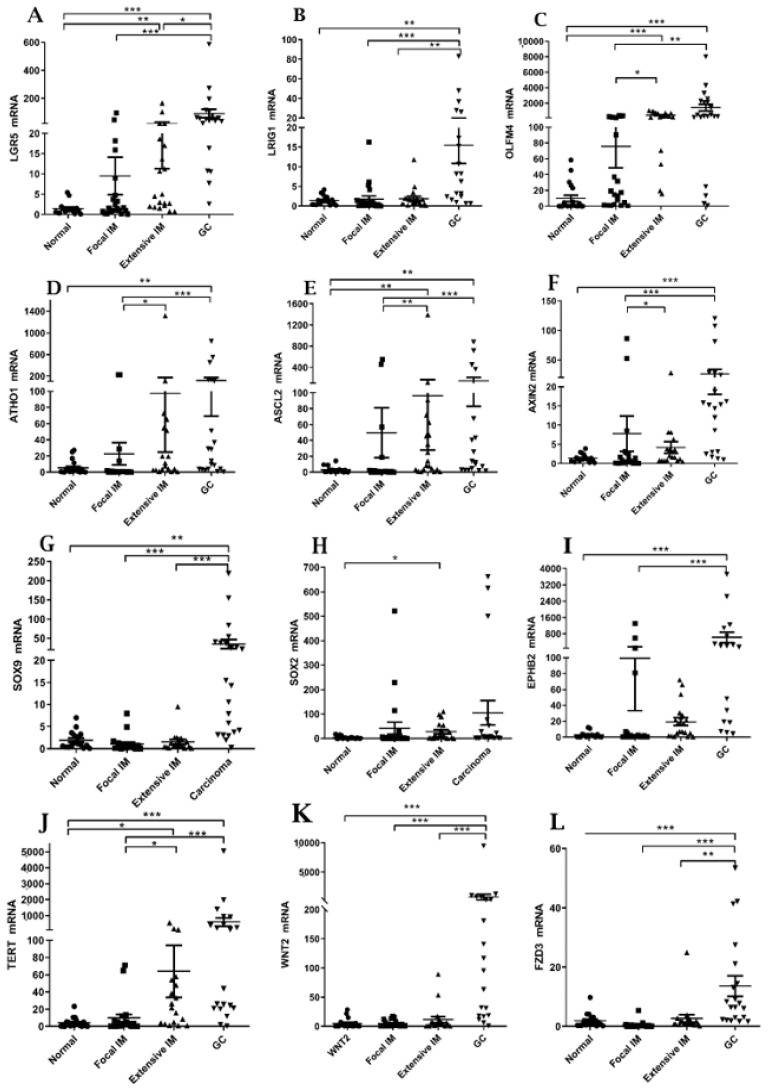
Expression levels of ISC marker genes in the four tissue categories. (**A**-**L**) Summary of qPCR quantification of gene transcripts. * *p* < 0.05; ** *p* < 0.005; *** *p* < 0.001. Mean ± SEM.

Intestinal and gastric stem cell markers characterize tissue resident stem cells that play a role in tissue homeostasis and repair and are dysregulated with the transition to malignancy [11]. We examined the relationship between *POPDC1* and *POPDC3* expression and the expression of twelve ISC markers. Compared with normal tissues, all twelve genes were significantly upregulated in the GC tissues, some (*LGR5, OLFM4, TERT*) were elevated in extensive IM and few (*LGR5, OLFM4*) were elevated in focal IM as well. (Figure 7). Table 2 lists the results of correlation analyses. The transcript levels of the twelve genes were inversely correlated with *POPDC1* expression although in the case of *LRIG1* and *WNT2* the correlation was not statistically significant. Regarding correlation with *POPDC3* expression, only six out of twelve ISC markers showed an inverse correlation with statistical significance (Table 2).

## 3. Discussion

We report the dysregulated expression of *POPDC* genes in human IM and GC tissues as evaluated in endoscopic gastric biopsies. Our results demonstrate, for the first time, the reduced expression of *POPDC1* and *POPDC3* in precancerous IM lesions and confirm previously reported downregulation of these genes in GC. Several genes formerly identified in relation to IM development and GC progression were co-assessed for comparison and were found to be regulated in a manner opposite to *POPDC* genes. The study, conducted in archival pathologically diagnosed samples, provides new insights as to the possible involvement of *POPDC1* and *POPDC3* in IM transdifferentiation and GC progression.

IM is primarily a histologic definition. In this study, the pathological analysis was based on H&E staining with no routine AB/PAS staining including the classification into focal and extensive IM. Although we did not group our IM samples according to the new guidelines on the management of IM published by Gupta and colleagues in 2020 [37], it is noteworthy that the pathological-histologic IM classification corresponded satisfactorily, though not completely, to the clustering of tissue samples by the bi-directional multi-transcript heatmap analysis. The tissues diagnosed as focal IM were segregated from tissues identified as extensive IM with little intermingling. This points to distinct transcriptomic pattern of each IM category and the potential use of a multitranscript approach to improve IM characterization.

To the best of our knowledge, the positive immunolabeling of *POPDC3*, not *POPDC1*, was reported in IM only once and was not extended to a larger IM cohort as we did in the current study [21]. *POPDC2* expression was essentially unchanged in the IM and GC specimens that corroborated previous reports in GC tissues and GC cell lines, suggesting that the regulation of *POPDC2* expression differs from that of *POPDC1* and *POPDC3* [21]. The co-regulation of *POPDC1* and *POPDC3* may result from their co-localization on the same chromosome 6q21, whereas *POPDC2* localizes to chromosome 3q13 [38].

The silencing of *POPDC1* and *POPDC3* during tumorigenic transformation is attributed primarily to promoter hypermethylation [21,39]. Other mechanisms reported to reduce *POPDC1* and *POPDC3* expression include histone de-acetylation, EGF signaling, and *AKT* activation by *netrin-1* [21,28,40,41]. We believe that the same mechanisms may underlie *POPDC1* and *POPDC3* downregulation in precancerous IM, yet additional studies are required to confirm this hypothesis.

Studies on *POPDC1* (BVES), the prototype of the *POPDC* family, have provided insights as to mechanisms through which *POPDC1* silencing or suppression facilitate tumor development and progression [14,15,18,29,30,42]. *POPDC1* maintains junctional structures and cell adhesion and suppression of *POPDC1* expression enhanced EMT and cell mobility. *POPDC1* re-expression reverted EMT and established cell contacts in cultured epithelial cells [20]. Likewise, in GC cell lines, the re-expression of POPDC3 reduced cell migratory and invasive capabilities [21,43]. Besides, *POPDC1* modulates cell shape and motility as well as vesicle trafficking that may also contribute to cancer cell transformation and tumor progression [21,44,45]. Both, *POPDC1* and *POPDC3* showed predominant cytoplasmic localization in tumors, suggesting that altered subcellular localization also plays a role in addition to differences in expression levels [22]. Furthermore, the interaction of *POPDC1* with molecules in pathways regulating cell proliferation and stem cell activation, such as *ZO-1*, *Wnt*, and *c-Myc* supports the role of *POPDC1* as a negative regulator of cell proliferation [20,23].

As depicted in the heatmap, the downregulation of *POPDC1* and *POPDC3* expression in IM concurred with increased expression of genes encoding transcription factors, mucins, and ISC markers previously associated with IM and GC development [46]. Correlation analyses between the transcript levels of *POPDC1* and *POPDC3* and a selection of these genes, demonstrated statistically significant inverse correlation with the majority of the genes. The elevated expression of *CDX2* appears already in focal IM, reflecting the transdifferentiation to intestinal phenotype. However, a pronounced c-Myc upregulation is evident mainly in the GC tissues and less in focal or extensive IM. Nonetheless, elevation in c-Myc protein may take place independently of c-Myc transcript upregulation since the enhancing effect of *POPDC1* on c-Myc degradation should be reduced when *POPDC1* is underexpressed, a condition expected in the extensive IM tissues [23].

Alterations in mucins, the mucosa protecting glycoproteins [47], are regarded as indicators of IM and premalignant transformation in the gastric mucosa [34,35]. Mucin expression levels and mucin glycosylation patterns (such as *MUC1, MUC2, MUC3, MUC4, MUC 5AC, MUC5B, MUC13*, and *MUC17*) reflect inflammation and transdifferentiation of normal gastric mucosa into intestinal phenotype [46]. We found inverse correlation between the expression of mucins that characterize severity of malignant transformation, and the tumor suppressors *POPDC1* and *POPDC3,* suggesting an inverse regulatory linkage.

We report the increased expression of ISC markers in IM and GC tissues that was inversely correlated with *POPDC1* and *POPDC3* expression. The observation that Wnt target gene and ISC marker *LGR5* is upregulated in IM tissues along with increased expression of ISC markers such as *OLFM4* and *EPHB2*, supports previous reports that indicate intestinal-like stem cell population as players in IM pathogenesis [11]. Increased ISC marker activation including amplified Wnt signaling and elevated expression of stem cell markers *LGR5* and *ASCL2* were reported in enteroids of *POPDC1*-depleted mice [25]. We postulate that *POPDC1* and *POPDC3* downregulation in gastric IM triggers the induction of stem cell markers and stem cell activation that facilitates IM transdifferentiation and GC progression.

Collectively, our results demonstrate the downregulation of *POPDC1* and *POPDC3* in focal and extensive IM that coincides with the upregulation of genes responsible for the development and maintenance of IM and the progression to GC. The association of *POPDC1* and *POPDC3* downregulation with IM and GC suggests a role in tumor suppression and highlights them as potential biomarkers for IM and GC progression and as prospective treatment targets.

Study limitations: This is an analysis of archived clinical pathology specimens. No systematic follow-up biopsies were available, neither adjacent samples of normal tissues. Therefore, the information obtained is essentially descriptive. Any mechanistic deduction inferred from the data is based on knowledge derived from the relevant literature. Whether the downregulation of *POPDC1* and *POPDC3* plays an active role controlling gene expression or is it a concomitant phenomenon awaits future investigation. Experiments in GC cell lines manipulated for *POPDC1* and *POPDC3* re-expression and over-expression may offer an answer. The information presented by us provides a basis and opens the way for prospectively designed investigation of human biopsies as well as experiments in animal models and cell cultures to clarify the role of *POPDC1* and *POPDC3* in IM transdifferentiation and GC progression.

## 4. Materials and Methods

### 4.1. Biopsy Selection

The study was approved by the Rabin Medical center institutional Ethics Committee (#RMC 027812). Endoscopic biopsies have been taken according to the clinical indications. Consecutive Formalin-fixed and paraffin-embedded (FFPE) gastric samples with or without intestinal metaplasia or cancer were collected from the pathology archive of Rabin Medical Center, from patients who underwent endoscopic gastric biopsies at the Gastroenterology Division of Rabin Medical Center, from 2005 to 2017. Normal tissue, IM or GC lesions, were determined by using H&E, Alcian Blue/PAS, and Giemza staining for *Helicobacter pylori*. The evaluation was done by two expert gastro-pathologists. IM lesions were categorized into focal and extensive IM. We excluded patients with *Helicobacter pylori* infection and patients with family history of gastric cancer. After exclusion, 80 biopsies were included in the study. The medical records of the patients were reviewed retrospectively, including flow-up after endoscopic procedures, gastric cancer staging, grading, and prognosis.

### 4.2. Sample Processing for Histochemistry and IHC

Three serial micro-sections (4 µm) were prepared from each FFPE sample and processed for either histochemistry and immunohistochemistry or the isolation of RNA. Slides were deparaffinized with xylene, and washed with serial dilutions of ethanol. For mucin staining we used a commercial Alcian Blue 2.5PH/PAS Stain kit (Bio-optica Milano Italy) according to the manufacture’s protocol including serial staining and washing steps. For immunohistochemistry, we employed heat-induced epitope retrieval methods with citrate buffer, pH 6.0 (#ab93678 Abcam, UK). POPDC1 was detected using a 1:100 dilution of a mouse monoclonal antibody (anti BVES (POPDC1), #sc-374081, Santa Cruz Biotechnology), POPDC3 was detected in parallel sections using a 1:100 dilution of a Rabbit polyclonal anti-POPDC3 antibody (#ab76388 Abcam, UK). Incubation with the primary antibodies was overnight at 4 °C. Bound antibodies were detected using HRP Polymer Detection System, ZytoChem Plus (HRP) One-Step Polymer anti-Mouse/Rabbit (#ZUC053-00, Zytomed Systems, Germany) and DAB Substrate Kit (ab64238 Abcam, UK), according to the manufactures protocol. Hematoxylin (blue) (Sigma-Aldrich) was used for counter staining. Of notice, preliminary experiments with several anti-POPDC2 antibodies did not yield satisfactory labeling and no IHC results were obtained for POPDC2. Photographs were taken at X20 magnification. Two expert pathologists examined the histological preparations.

### 4.3. RNA Isolation and Quantitative Real-Time PCR (qPCR)

RNA was isolated from FFPE tissue sections using RNAeasy mini columns (Qiagen, Valencia, CA, USA). The manufacturer’s protocol was followed with the exception of increased proteinase K digestion time (overnight incubation). RNA quantity and quality were determined by OD determination at 260 and 280 nm using a Nano Drop spectrophotometer (Nano Drop Technologies, Wilmington, DE, USA). RNA was converted to cDNA using Revert Aid First Strand cDNA Synthesis Kit (Fermentas, Thermo Fisher Scientific, USA) and was quantified by qPCR, performed as described previously [48]. We used the TaqMan and or SYBR green system with pre-amplification employing PreAmp Master Mix kit (Applied Biosystems, Thermo Fisher Scientific, USA) to target genes. Fluorescence accumulation was analyzed by StepOnePlus Real-Time PCR System (Applied Biosystems, Thermo Fisher Scientific, USA). For accuracy, gene expression was normalized to three endogenous control genes: *18S ribosomal RNA, RPLP0,* and *HTRP1*. The results reported here are based on normalization to *RPLP0*, the most consistently expressed housekeeping gene. The details of SYBR green primer-pairs and TaqMan assays from IDT DNA Technologies USA are listed below:

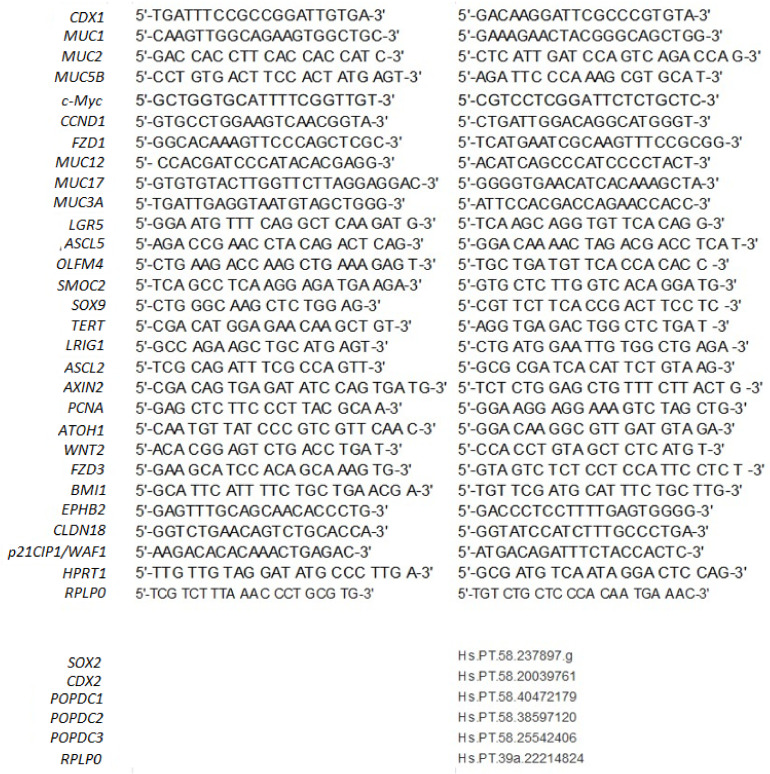



### 4.4. Cell Culture

Human GC cell lines SNU-16 (ATCC CRL-5974) and SNU-719 (CVCL_5086) were obtained from Korea Cell Line Bank (Seoul, Korea) and NCI-N87 from the ATCC (ATCC CRL-5822). Human embryonic kidney 293T cell line was from the ATCC (ATCC CRL-3216). Cells were grown in DMEM supplemented with 10–15% fetal bovine serum (Biological Industries, Israel) and maintained at 37 °C in a humidified atmosphere with 5% CO2.

### 4.5. Statistical Analysis

Comparison between groups was performed using ANOVA, Mann–Whitney–Wilcoxon test and two-tailed Student’s *t*-test for independent data. Spearman’s rank correlation was calculated using GraphPad software and JMP pro13 software. *p* < 0.05 was considered significant.

## Figures and Tables

**Table 1:** Demographic details of the study population.

	Normal	Gastric IM		GC
		Focal IM	Extensive IM	
Number	20	22	18	20
Age (Years ± SD)	69.5 ± 9.9	69.6 ± 10.1	66.7 ± 11.2	76.8 ± 9.9
Males (*N* %)	9 (45%)	12 (55%)	7 (39%)	10 (50%)
Females (*N* %)	11 (55%)	10 (45%)	11 (61%)	10 (50%)

IM = intestinal metaplasia; GC = gastric cancer.

**Table 2:** Summary of correlation analyses between transcript levels of *POPDC1* and *POPDC3* and those of the genes depicted in Figure 7. Spearman’s correlation test.

*POPDC1*		*POPDC3*		
	R	P	r	p
*LGR5*	−0.443	<0.0001	−0.302	<0.05
*LRIG1*	−0.164	NS	0.114	NS
*OLFM4*	−0.593	<0.0001	−0.521	<0.01
*ATHO1*	−0.359	<0.005	−0.277	<0.05
*ASCL2*	−0.373	<0.005	−0.297	<0.05
*AXIN2*	−0.323	<0.01	−0.185	NS
*SOX9*	−0.348	<0.005	−0.018	NS
*SOX2*	−0.256	<0.05	−0.235	NS
*EPHB2*	−0.471	<0.0001	−0.361	<0.01
*TRET*	−0.387	<0.001	−0.256	<0.05
*WNT2*	−0.229	NS	−0.129	NS
FZD3	−0.26	<0.05	0.063	NS

## Data Availability

Data supporting the reported results can be obtained from The Felsenstein Medical Research Center, Rabin Medical Center, Petah Tikva, upon demand.
